# Bladder rupture 11 years after partial cystectomy for bladder endometriosis: A case report and review of literature

**DOI:** 10.1016/j.crwh.2024.e00657

**Published:** 2024-10-16

**Authors:** Haruna Tokuyama, Yosuke Tarumi, Saiko Yamauchi, Hiroyuki Okimura, Hisashi Kataoka, Tetsuya Kokabu, Kaori Yoriki, Fumitake Ito, Izumi Kusuki, Taisuke Mori

**Affiliations:** a Department of Obstetrics and Gynecology, Kyoto Prefectural University of Medicine, Graduate School of Medical Science, Kyoto, Japan; b Department of Obstetrics and Gynecology, Kyoto Yamashiro General Medical Center, Kyoto, Japan

**Keywords:** Deep endometriosis, Laparoscopic surgery, Complication, Bladder rupture, pseudo-renal failure

## Abstract

Partial cystectomy is often performed to treat bladder endometriosis. However, there are no reports of bladder rupture more than 10 years after cystectomy. A 55-year-old woman with a history of laparoscopic bilateral salpingo-oophorectomy and partial cystectomy for bladder endometriosis at the age of 44 years presented with worsening dysuria, decreased urine output, and malaise for over a week. Blood tests revealed elevated creatinine and BUN levels indicating renal failure. Transvaginal ultrasonography and computed tomography revealed large amounts of peritoneal fluid. Abdominocentesis was performed, and peritoneal fluid analysis confirmed the presence of urinary ascites, which was indicative of bladder rupture. Retrograde cystography revealed contrast leakage into the bladder wall. Therefore, a diagnosis was made of with bladder rupture and pseudo-renal failure. If abdominal pain and peritoneal fluid are present after bladder endometriosis surgery, bladder rupture should be considered in the differential diagnosis even after a long postoperative period.

## Highlights

• Partial cystectomy is often performed to treat bladder endometriosis.
• No study has reported complications of bladder rupture a long time after cystectomy.
• The in this case presented with pseudo-renal failure and bladder rupture 11 years after cystectomy.
• Bladder rupture should be considered in a differential diagnosis even after a long postoperative period.

## 1 Introduction

Endometriosis, a chronic inflammatory disease affecting 10 % of women of reproductive age, is categorized into three types: peritoneal, ovarian, and deep endometriosis (DE) [1]. Bladder endometriosis, accounting for 11 % of DE lesions, causes lower urinary tract symptoms, including dysuria, hematuria, bladder pain, and urgency. Although partial cystectomy is generally a safe and simple procedure, complications like postoperative bladder rupture can occur [2,3]. Bladder rupture is defined as the leakage of urine into the abdominal cavity due to trauma or other factors. Approximately 96.6 % of bladder ruptures are caused by trauma and spontaneous ruptures are rare. As symptoms are often nonspecific, such as lower abdominal pain, and the mortality rate due to spontaneous rupture is significant (15–80 %) [4], a careful and prompt diagnosis is needed. Postoperative bladder rupture after partial cystectomy for bladder endometriosis has been documented [3], but none more than 10 years after surgery. Herein, a rare case of bladder rupture 11 years after surgery for bladder endometriosis is reported and the related literature is reviewed.

## 2 Case Presentation

**Fig. 1:**
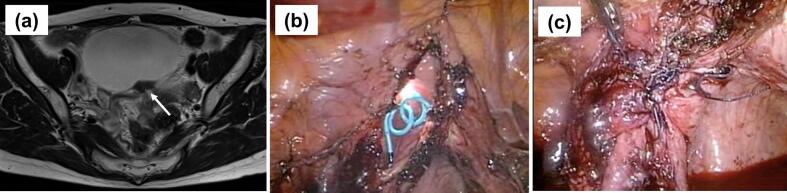
MRI and intraoperative findings of partial cystectomy for bladder endometriosis at the age of 44 years. (a) MRI T2-weighted image shows a low-signal nodule on the dorsal surface of the bladder, which was thought to be an endometriotic lesion (arrow). (b) The endometriosis lesion and bladder mucosa were resected by partial cystectomy. (c) A two-layer suture was administered in the bladder wall. The bladder mucosal surface was continuously sutured with 2–0 vicryl, followed by repair of the muscular layer with a single suture.

The patient was a 55-year-old woman (gravida 3, para 3) with a history of total laparoscopic hysterectomy for uterine leiomyoma at the age of 43 years and laparoscopic bilateral salpingo-oophorectomy and partial cystectomy for bladder endometriosis at the age of 44 years. A 23 × 11 × 43 mm nodule on the posterior wall of the bladder was completely resected by partial cystectomy using laparoscopy and cystoscopy (Figs. 1a, b). The bladder wall was repaired using a two-layer suture with 2–0 vicryl for the mucosal and muscular layers (Fig. 1c).

The patient was followed up at the hospital for 11 years. At the age of 55 years, she had frequent urination accompanied by pain and was diagnosed with cystitis by her local physician; the symptoms improved after a three-week course of an antibacterial agent before an emergency visit to the hospital. One week prior, she experienced severe pain in her lower abdomen and visited the hospital with the chief complaints of worsening dysuria, decreased urine output, and malaise.

**Fig. 2:**
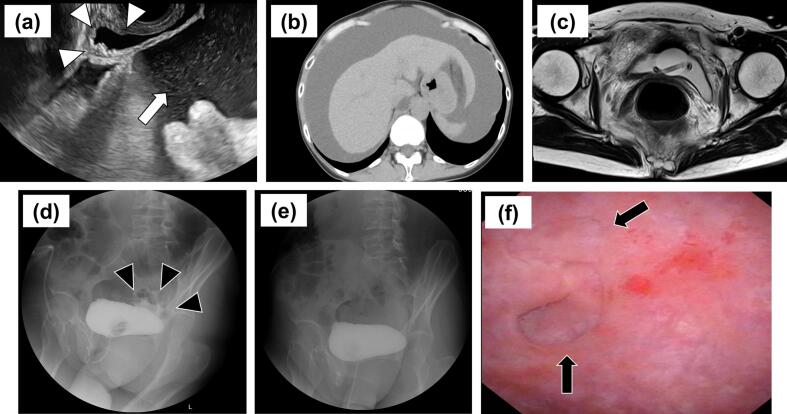
Imaging findings of bladder rupture at the age of 55 years. (a) Transvaginal ultrasonography shows a moderate amount of urine retention in the bladder (white triangles) and a large amount of peritoneal fluid (white arrow). (b) Computed tomography shows peritoneal fluid from below the diaphragm to the pelvic cavity. (c) MRI T2-weighted image shows no lesion of endometriosis in the bladder. (d) Retrograde cystography shows minor contrast leakage from the posterior wall of the apex of the bladder (black triangles). (e) Cystogram on day 9 after admission shows no contrast leakage. (f) On day 10, cystoscopy shows bladder diverticulum caused by previous cystectomy (black arrows).

Transvaginal ultrasonography revealed moderate retention in the bladder and a large amount of peritoneal fluid (Fig. 2a). No bladder wall disruption was observed. Blood analysis revealed elevated creatinine (4.41 mg/dL), blood urea nitrogen (BUN) (56.6 mg/dL), and K (5.6 mEq/L) levels and decreased Na (132 mEq/L) levels. Computed tomography showed peritoneal fluid extending from below the diaphragm to the pelvic cavity (Fig. 2b). Magnetic resonance imaging (MRI) confirmed the absence of endometriotic lesions (Fig. 2c).

Based on a history of partial resection of the bladder wall, severe renal dysfunction, and ascites, bladder rupture was suspected (11 years after the bladder cystectomy). Following abdominocentesis, 3100 mL of serous and yellow fluid was drained, which on analysis confirmed urinary ascites (creatinine, 15.48 mg/dL; BUN, 99.0 mg/dL; K, 8.9 U/L). Retrograde cystography revealed minor contrast leakage from the posterior bladder wall (Fig. 2d). Accordingly, the patient was diagnosed with bladder rupture. As the leakage was minor, conservative management was planned, and a urinary catheter was placed in the bladder. The abdominal pain gradually decreased after admission. On the second day, levels of creatinine and BUN (0.71 and 19.9 mg/dL, respectively) decreased. On day 9, retrograde cystography confirmed the absence of contrast leakage (Fig. 2e), and on day 10, cystoscopy confirmed a bladder diverticulum caused by a previous cystectomy and normal ureteral opening with no fistula site (Fig. 2f). On day 11, the catheter was removed and no ascites retention was observed. The patient was discharged on day 12. No recurrence of symptoms or bladder rupture was noted over two years.

## 3 Discussion

The treatment of bladder endometriosis includes hormonal and surgical therapies. Hormonal treatment – including low-dose estrogen and progestin, progestogens (LEP), and gonadotropin-releasing hormone (GnRH) analogs – being efficacious, safe, and well tolerated, is generally the first-line therapy [2,3]. A study on bladder endometriosis reported symptom improvement in 92 % of the patients receiving hormonal therapy [5]. A study that included 15 patients who received hormonal therapy (some of whom switched treatments) reported that dienogest, LEP, and GnRH analogs were effective and tolerable in 9 of 10 (90.0 %), 5 of 9 (55.6 %), and 2 of 3 patients (66.7 %), respectively [6]. Alternatively, surgical management, such as transurethral resection and partial cystectomy (alone or in combination), is selected for patients who develop the disease during treatment. Furthermore, significant improvements in pain and urinary symptoms and minimization of recurrence risk have been reported [2]. However, no detailed studies on cystectomy-related complications are available.

To investigate the complications of partial cystectomy, studies that included more than 10 cases of partial cystectomy for bladder endometriosis (Table 1) were reviewed [3,[7], [8], [9], [10], [11], [12], [13]]. A total of 492 cases from eight studies were included: one case was not indicated for surgery [11] and 10 were administered only shaving or unnecessary resection of the bladder wall [13]; partial cystectomy was performed in the remaining 481 patients, via laparoscopy in 439 cases (91.3 %) and laparotomy in 42 (8.7 %). The suture string and method varied between braided and monofilament and between single and two layers in each study. The mean follow-up period was 12–44 months. One study involving 69 patients did not include data on complications [12]. Postoperative complications occurred in 47 of the 412 patients (11.4 %), with a frequency ranging from 0 to 17.4 %. The most frequent complications were peritonitis, pelvic abscess, and postoperative fever in 12 patients (2.9 %) and bowel-related complications in 9 patients (2.2 %). Bladder rupture, including urinary leakage and bladder fistula, was reported in 7 cases (1.7 %). No cases of bladder rupture occurring more than 10 years after partial cystectomy have been reported.

**Table 1:** LPS, laparoscopic; PC, partial cystectomy. Data is represented as mean (range), mean ± standard deviation, or range alone.

No.	Reference	Number of cases	Age, year	Size of nodule, mm	Operative procedure, number	Suture string	Suture method	Follow-up period, month	Postoperative complication, number	Details
1	Nezhat et al. 2002 [9]	15	37(27–57)	28 (4–55)	LPS PC	–	Single-layer, *n* = 14 Two-layer, *n* = 1	–	2 (13.3 %)	Extravasation without leakage into the peritoneum, *n* = 2
2	Fedele et al. 2005 [10]	47	30.5 ± 3.2	–	PC, *n* = 29 LPS PC, *n* = 18	3–0 braided absorbable string (Vicryl)	Two-layer	33.5 (24–108)	8 (17.0 %)	Postoperative fever, *n* = 8
3	Salvatores et al., 2007 [11]	21	32 (25–40)	29.2 (10–50)	LPS PC	–	–	19 (5–27)	2 (9.5 %)	Peritonitis, n = 1 Fistula between bowel and bladder, n = 1
4	Litta et al. 2012 [12]	12	33–48	20–35	LPS PC	3–0 monofilament string	Continuous two-layer	more than 12	0 (0 %)	(Suprapubic pelvic pain, n = 3)
5	Kjer et al. 2014 [13]	31	30.3 (23–44)	27 (12–40)	PC, *n* = 13 LPS PC, *n* = 17 Surgery not yet indicated, n = 1	2–0 braided absorbable string (Vicryl)	Single- or two-layer	44 (13–125)	5 (16.1 %)	Minor urinary leakage, n = 2 Major urinary leakage, n = 1 Compartment syndrome, n = 1 Ogilvies syndrome, n = 1
6	Soriano et al. 2016 [14]	69	31.3 ± 4.5	28.6 ± 6.0, 17.8 ± 7.7 (two groups)	LPS PC	3–0 monofilament absorbable string	Single-layer	more than 36	–	–
7	Ceccaroni et al. 2020 [5]	264	36.8 ± 5.6	32.5 ± 13.0	LPS PC	3–0 monofilament absorbable string (Monocryl)	Two-layer	more than 12	26 (9.8 %)	*Within 28 days after surgery, *n* = 19 **Over 28 days after surgery, n = 7
8	Piriyev et al. 2023 [15]	33	35.0 ± 6.3	25 ± 10	LPS PC, *n* = 23 LPS shaving, *n* = 7 No resection, n = 3	Braided absorbable string (Vicryl, n = 18) Monofilament absorbable string (V-Loc, *n* = 5. PDS, *n* = 3)	–	–	4 (4/23, 17.4 %)	Postoperative bleedng, n = 3 Ureteral stenosis, n = 1

Detailed data of *: Vesico-vaginal fistula, n = 2. Vesical fistula, n = 1. Intravesical bleeding, *n* = 2. Uroperitoneum, *n* = 1. Hemoperitoneum, *n* = 4. Bowel-related complications, *n* = 6. Pelvic abscess, n = 3. Blood transfusion, n = 5 (There is overlap of complications). **: Ureteral fistula, n = 1. Ureteral stenosis, *n* = 3. Bowel-related complications, n = 3.

Most cases of bladder rupture are secondary to bladder injury and trauma, including previous bladder surgery. In 3.4 % of bladder rupture cases, there was no history of injury or trauma, referred to as spontaneous rupture of the urinary bladder (SRUB) [4]. Factors associated with SRUB include pelvic radiation, alcohol intoxication, urinary tract infection, inflammation, malignant disease, and neurogenic bladder, such as previous cerebrovascular events, diabetes mellitus, or multiple sclerosis [4,14]. Bladder endometriosis is a risk factor for SRUB [14]. In this case, inflammation within the bladder due to urinary infection was associated with bladder rupture, in addition to weakness of the bladder wall due to a cystectomy 11 years previously. No bladder endometriotic lesions were confirmed on MRI.

Owing to its high mortality rates, bladder rupture warrants prompt diagnosis and treatment. In a recent review of 278 articles, the overall mortality rate was 15 %, and most deaths occurred within 72 h of presentation. Furthermore, 3.7 % of patients die before the correct diagnosis or intervention [4]. Symptoms include lower abdominal pain associated with dysuria, an inability to void, anuria, and hematuria [15]. Biochemical examinations are useful in detecting “pseudo-renal failure” [4], characterized by the transfer of small molecules, including creatinine and BUN, from the uroperitoneum across the peritoneal membrane into the blood, resulting in an increase in serum creatinine and BUN levels. These results may be misinterpreted as renal failure and further confounded by the presence of oliguria or anuria, prompting the initiation of renal replacement therapy. If the bladder wall defect is repaired or bladder drainage is performed, creatinine and BUN levels generally resolve within 24 h [4]. Retrograde cystography shows higher specificity (96 %) than CT without cystography (61 %) in the diagnosis of traumatic bladder rupture. However, this approach has recently been shifted to CT cystography because of its comparable specificity and lower invasiveness [4,15]. In the present case, bladder rupture was suspected based on the symptoms of lower abdominal pain, dysuria, and yellow and serous ascites, indicating urine contamination. Elevated creatinine and BUN levels led to a diagnosis of bladder rupture using retrograde cystography.

This is a rare case of uterine rupture occurring more than 10 years after surgery for bladder endometriosis. With the occurrence of abdominal pain and peritoneal ascites long after bladder endometriosis surgery, the possibility of bladder rupture should still be considered.
